# Modulation of Gut Microbiota and Antibiotic Resistance Genes by Heat-Killed *Enterococcus faecalis* EF-2001 in High-Fat Diet-Induced Obesity Mice: A Shotgun Metagenomics Study

**DOI:** 10.3390/bioengineering12070741

**Published:** 2025-07-07

**Authors:** Ranjith Kumar Manoharan, Kwon-Il Han, Hyun-Dong Shin, Yura Lee, Sunhwa Baek, Eunjung Moon, Youn Bum Park, Junhui Cho, Sathiyaraj Srinivasan

**Affiliations:** 1 Research & Development Center, Bereum Co., Ltd., Wonju 26361, Republic of Korea; kihan@bereum.com (K.-I.H.); hshin@bereum.com (H.-D.S.); lyr@bereum.com (Y.L.); an20112246@bereum.com (S.B.); moonej@bereum.com (E.M.); pyb12345@bereum.com (Y.B.P.); jhcho@bereum.com (J.C.); 2 Department of Bio & Environmental Technology, College of Natural Science, Seoul Women’s University, 623 Hwarangno, Nowon-gu, Seoul 01797, Republic of Korea

**Keywords:** *Enterococcus faecalis* EF-2001, postbiotics, high-fat diet, obesity, metagenomics

## Abstract

The gut microbiome is vital in maintaining metabolic health, and dietary habits can significantly impact its composition. A high-fat diet (HFD) can disrupt gut microbial balance, contributing to obesity, insulin resistance, and fatty liver disease. This study explores the potential benefits of heat-killed *Enterococcus faecalis* EF-2001 (EF-2001) in restoring gut balance and improving metabolic health in HFD-fed mice (HFD-mice). HFD mice administered EF-2001 had 18% less body fat, 22% lower triglyceride levels, and significantly reduced liver enzyme markers, including aspartate aminotransferase (AST) by 28% and alanine aminotransferase (ALT) by 31%. Additionally, EF-2001 improved glucose metabolism, increasing glucose tolerance by 20% and insulin sensitivity by 15%, while reducing fat buildup in the liver by 24%, indicating protection against fatty liver disease. These changes correlated with better metabolic health and reduced inflammation. Our results show that EF-2001 supplementation helped counteract HFD-induced gut imbalances by increasing microbial diversity and supporting beneficial bacteria, such as *Akkermansia* and *Ligilactobacillus* spp. Our findings highlight the potential of heat-killed EF-2001 as a promising strategy to restore gut balance and mitigate diet-related metabolic issues. Furthermore, analysis of antibiotic resistance genes (ARGs) revealed that HFD mice exhibited an increased abundance of multidrug resistance genes, particularly those associated with antibiotic efflux mechanisms, such as *bcr*A, *cde*A, and *msb*A. Notably, EF-2001 supplementation mitigated this increase, reducing the relative abundance of the above ARGs and suggesting a protective role in limiting the spread of antibiotic resistance linked to dysbiosis. EF-2001 offers a compelling approach to managing obesity and metabolic disorders, paving the way for microbiome-based health interventions.

## 1. Introduction

Obesity has emerged as a global health crisis, significantly increasing the risk of chronic diseases, such as type 2 diabetes, cardiovascular disorders, and metabolic syndrome. According to the World Health Organization (WHO), obesity rates have nearly tripled worldwide since 1975, underscoring the urgent need for effective prevention and treatment strategies [1]. The gut microbiota has garnered increasing attention for its critical role in metabolic regulation and energy balance [2].

The gut microbiome, a complex ecosystem of trillions of microorganisms, plays a fundamental role in host metabolism, immune function, and overall health [3]. Emerging research indicates that gut dysbiosis, an imbalance in the microbial community, may be a potential contributing factor to obesity, alongside well-established drivers such as dietary habits and physical activity [4]. Studies have shown that individuals with obesity exhibit distinct gut microbiota profiles, often characterized by an increased Firmicutes-to-Bacteroidetes ratio, altered short-chain fatty acid (SCFA) production, and an enhanced capacity for energy extraction from food [5,6]. Certain gut bacteria, such as *Eubacterium rectale* and *Clostridium coccoides*, have been correlated with obesity phenotypes [7]. This dysbiotic microbial environment is associated with chronic low-grade inflammation, lipid accumulation, and insulin resistance, contributing to obesity pathogenesis [8]. Advances in metagenomic sequencing have provided more profound insights into gut microbial diversity, shedding light on how microbial alterations influence metabolic health and dietary responses.

Given the critical role of the gut microbiota in obesity, strategies aimed at modulating microbial composition have gained significant interest. Probiotics are live microorganisms that, when administered in adequate amounts, confer health benefits on the host [9]. They play a crucial role in modulating the gut microbiota, strengthening the intestinal barrier, and interacting with the host immune system [10]. Various probiotic strains, including Lactobacillus, Bifidobacterium, and Enterococcus, have demonstrated beneficial effects by strengthening gut barrier integrity, reducing inflammation, and regulating lipid metabolism [11]. For instance, *Lacticaseibacillus paracasei* AO356 significantly altered the gut microbiota composition in a mouse model, suppressing weight gain and fat mass accumulation [12]. This strain increased the relative abundance of Bacteroides and Oscillospira, which are closely linked to lipid metabolism and obesity-related markers [12]. Another study involving *L. paracasei* K56 showed improvements in stress, anxiety, and sleep quality in students, along with beneficial changes in gut microbiota composition, suggesting potential broader health benefits that could indirectly impact obesity-related behaviors [13]. In addition, probiotic *Enterococcus faecalis* EF-1 has been shown to mitigate high-fat diet (HFD)-induced obesity through its cholesterol-lowering properties, bile salt hydrolase activity, and ability to enhance SCFA production [14]. In a study on geese, *E. faecalis* supplementation increased body weight, reduced abdominal fat and hepatic lipid droplet content, and significantly decreased serum levels of total cholesterol, triglycerides, and free fatty acids, consequently positively impacting hepatic lipid metabolism-related genes and improving ileal morphology and microbiota diversity [15]. These findings indicate that specific strains of *E. faecalis* hold promise as potential therapeutics or functional food components for managing obesity and related metabolic disorders. However, the clinical application of probiotics faces challenges related to strain viability, stability, and efficacy [16,17]. To address these limitations, postbiotics, such as non-viable probiotics (whole-cell postbiotics), cellular components, or metabolites, have emerged as a novel alternative, offering similar health benefits without the need for live bacterial cultures [18]. Unlike probiotics, which require viability for colonization and some functional benefits, whole-cell postbiotics, including heat-killed probiotics, may exert health effects primarily through their structural components, such as cell wall polysaccharides, lipoteichoic acids, and peptidoglycans, which interact with host immune receptors to modulate immune responses and improve gut barrier function [19]. Their non-viable nature enhances safety and stability during processing and storage, addressing concerns associated with live microorganisms in immunocompromised individuals [20]. Recent investigations have shown that heat-killed probiotics can modulate obesity and the induced inflammation in HFD mice [21].

Postbiotics also refer to the bioactive metabolites produced by probiotics during fermentation. These include SCFAs, antimicrobial peptides, cell wall fragments, and other biologically active compounds that can benefit the host without live microorganisms [22,23]. Postbiotics are particularly valuable because of their stability during processing and storage, making them attractive candidates for functional food applications and therapeutic interventions. Although certain strain-specific exopolysaccharides (EPSs) have been reported to possess antimicrobial properties, EPSs are more commonly known for their protective roles, including biofilm formation and shielding bacteria from antibiotics and environmental stress [24]. In particular, whole-cell postbiotics, including heat-killed probiotics, offer a dual benefit by combining structural components with residual intracellular compounds, enabling broader immunomodulatory and gut health effects compared to metabolites alone [25]. Recent studies have demonstrated that postbiotics can suppress obesity by promoting thermogenesis and altering gut microbiota composition [26].

Our research group has explored the potential of heat-killed *E. faecalis* EF-2001 (EF-2001), a postbiotic with demonstrated immunomodulatory and metabolic benefits, in conditions associated with metabolic dysfunction. While *E. faecalis* strain EF-1 has been studied for its metabolic activities and general benefits to gut health, it has largely demonstrated conventional probiotic properties. In contrast, EF-2001, a heat-killed strain, has demonstrated more targeted efficacy, particularly in regulating metabolic pathways and improving outcomes in models of obesity and fatty liver disease. Notably, previous research suggests that EF-2001 may play a role in mitigating non-alcoholic fatty liver disease (NAFLD), a disorder closely linked to obesity [27]. By regulating lipid metabolism, EF-2001 may offer a dual advantage in managing obesity and its associated comorbidities, such as NAFLD. Evidence indicates that EF-2001 may reduce hepatic lipid accumulation and protect against high-fat diet (HFD)-induced fatty liver damage by activating lipolysis through the AMPK signaling pathway [28]. This activation leads to decreased fat mass, liver index, adipocyte area, and levels of total cholesterol (TC) and low-density lipoprotein (LDL) while increasing high-density lipoprotein (HDL) levels [28]. Previous research on *E. faecalis* strains suggests their ability to influence gut microbiota composition, reduce lipid accumulation, and improve glucose metabolism, making them promising candidates for obesity intervention [14]. For example, *Lactobacillus plantarum* postbiotics have been reported to activate AMPK-dependent autophagy to suppress Salmonella intracellular infection and modulate inflammatory responses via NLRP3 inflammasome inhibition [29]. Furthermore, postbiotics have been reported to enhance gut microbial diversity while selectively promoting beneficial bacteria, contributing to a healthier microbial ecosystem [14]. Recent research indicates that diet, especially an HFD, can dramatically change the makeup and functional ability of the gut microbiome, including the resistome (i.e., the group of ARGs) [30]. Specific ARG enrichment has been demonstrated to correlate with dysbiosis linked to obesity, maybe as a result of selective microbial proliferation under conditions of metabolic stress and inflammation brought on by an HFD [31]. Consequently, monitoring ARGs offers information about how postbiotic therapies and dietary changes may affect not just the makeup of microbiota but also microbial activities that are relevant to public health. In another study, Koorakula et al. [32] highlighted that dietary composition, especially an HFD, can significantly alter the gut microbial ecology and promote dysbiosis, which, in turn, influences the expression and dissemination of ARGs.

By examining the impact of *E. faecalis* EF-2001 postbiotics on gut microbiota composition, lipid metabolism, and inflammatory markers, this study seeks to provide valuable insights into the potential role of postbiotics as a safe and effective therapeutic approach for obesity. Metagenomic sequencing enables a comprehensive analysis of microbial shifts, shedding light on the underlying mechanisms by which postbiotics exert their beneficial effects. A deeper understanding of the interplay between the gut microbiota and metabolic health could pave the way for innovative interventions in managing obesity and its related disorders.

## 2. Materials and Methods

### 2.1. Animal Experimental Design

Seven-week-old male C57BL/6N mice were obtained from Orient Bio Inc. (Seongnam, Republic of Korea) and housed under standard laboratory conditions (22 ± 2 °C, 12-h light/dark cycle, 55 ± 15% humidity). The animals had ad libitum access to food and water and were acclimated for three weeks before the start of the experiment (Figure S1). Following acclimatization, the mice were randomly assigned to one of three groups: a normal diet (ND) group (*n* = 5 + 3), a high-fat diet (HFD) group (*n* = 10 + 3), and an HFD (*n* = 10 + 3) group supplemented with heat-killed *E. faecalis* EF-2001 (HFD-EF-2001) 30 billion cells/day, via oral administration.

The ND group was fed a standard rodent chow diet (2918C, Teklad, Envigo, Indianapolis, IN, USA). In contrast, the HFD and HFD-EF-2001 groups were fed a high-fat diet (D12492, Research Diets, New Brunswick, NJ, USA), which provided 60% kcal from fat (5.55% kcal soybean oil and 54.35% kcal lard). This study commenced once the mice reached a body weight exceeding 30 g. After three weeks of HFD feeding (diet-induced obesity, DIO), body weight and blood chemistry measurements were used to ensure uniform group allocation. At this point, an additional three mice from each obesity-induced group (HFD and HFD-EF-2001), as well as the ND group, were selected for blood collection and subsequently excluded from further physiological assessments, such as body weight monitoring, biochemical analyses, fecal collection, blood glucose measurement, and necropsy. The remaining 10 mice per group were used for all main analyses, and no animals were excluded after the experiment began.

All experimental procedures were approved by (C) Woojung Bio. Institutional Animal Care and Use Committee (IACUC, approval number: IACIC2403-005) and conducted at Hu-mic, Inc under ethical guidelines for the care and use of laboratory animals.

### 2.2. Body Weight, Blood Chemistry, and Organ Weight Measurements

Body weight was recorded twice weekly throughout the ten-week experimental period, totaling 20 measurements per animal. Food intake was monitored weekly, and cumulative dietary consumption was calculated accordingly.

Blood chemistry analyses, including alanine aminotransferase (ALT), aspartate aminotransferase (AST), and triglyceride (TG) levels, were performed at four key time points: prior to disease induction, before experimental diet administration, at six weeks of dietary intervention, and after study completion. Blood samples were collected biweekly from three mice per group and centrifuged at 3000 rpm for 20 min to isolate the serum, which was subsequently stored at −80 °C for further biochemical analysis. Fasting blood glucose levels were measured in all the remaining animals at the same four time points. Additionally, fecal samples were collected before disease induction, prior to dietary intervention, at six weeks, and at the end of the experiment. All fecal samples were stored at −70 °C for subsequent gut microbiome analysis.

Following the ten-week dietary intervention, all the surviving mice were euthanized under anesthesia using chloral hydrate (400 mg/kg BW), and necropsy was performed. Liver and adipose tissues (subcutaneous, gonadal, perirenal, mesenteric, and pericardial fat) were excised, weighed, and stored at −80 °C for further analysis.

### 2.3. Serum Assay for Biochemical Parameters

Blood samples were collected at designated time points for biochemical analysis. ALT, AST, and TG levels were measured with a Hitachi Automatic Analyzer 7600–210 (Hitachi, Tokyo, Japan) to evaluate liver function and lipid metabolism. Serum was separated via centrifugation and stored at −80 °C for subsequent analyses.

### 2.4. Fecal DNA Extraction and Quality Control

Fecal samples from each group were collected at three time points during the in vivo experiments: at 0, 6, and 10 weeks from the ND, HFD, and HFD-EF-2001 groups. The samples were placed into sterile 50 mL tubes and stored at −80 °C until DNA extraction was performed. DNA was extracted from the randomized samples using a QIAmp PowerFecal DNA Kit (Qiagen, Hilden, Germany), following the manufacturer’s instructions. DNA quality was assessed by measuring concentration (Qubit fluorometer), purity (NanoDrop spectrophotometer), and integrity (Agilent TapeStation). For sequencing, DNA was sonicated into ~200 bp fragments using a Bioruptor and purified with a QIAQuick PCR Clean-Up Kit before re-quantification.

### 2.5. Shotgun Metagenomics Sequencing

The genomic DNA samples from the ND (*n* = 5), HFD (*n* = 5), and HFD-EF-2001 (*n* = 5) groups were then subjected to shotgun metagenomic sequencing. DNA quantification was performed again using a Qubit^TM^ dsDNA HS Assay Kit (Thermo Fisher Scientific, Waltham, MA, USA) on a Qubit 3 fluorometer (Thermo Fisher Scientific, Waltham, MA, USA) to ensure accurate measurement of double-stranded DNA. Metagenome sequencing was carried out by Dx&Vx (Seoul, Korea). Metagenomic libraries were prepared and sequenced using the Illumina NovaSeq 6000 platform (Illumina, San Diego, CA USA), which utilizes next-generation sequencing (NGS) technology. The sequencing process involved clonal amplification and sequencing by synthesis (SBS), where fluorescent signals emitted during nucleotide incorporation were detected, providing high-throughput and accurate sequencing. The raw sequencing data were processed with Illumina’s Real-Time Analysis (RTA) software (RTA version 1.18.66.3; Illumina, Inc., San Diego, CA, USA) for base calling and image processing. The resulting base call (BCL/cBCL) files were then converted into FASTQ format using either bcl2fastq2 v2.20.0.422 or bclconvert v3.10.5 with default settings. The sequencing run generated paired-end reads with a read length of 150 bp, covering 51 DNA samples, which were later used for downstream bioinformatics analysis.

### 2.6. Gut Microbiota Analysis

For shotgun metagenomic sequence analysis and taxonomic profiling, we combined several tools, including Kraken2, Bracken, Centrifuge, and MetaPhlAn, to ensure accurate and detailed results [33]. We began by processing the raw sequencing reads with Kraken2, a fast classifier that uses k-mer analysis to assign taxonomy at various levels. To improve the accuracy of the taxonomic abundance estimates and address any potential biases from Kraken2, we used Bracken, which refined the abundance calculations. To further validate and enhance the taxonomic resolution, we used Centrifuge, another classification tool that compared the reads against a comprehensive microbial genome database. Finally, we used MetaPhlAn to profile the microbial communities more precisely by focusing on specific marker genes, which helped us uncover both dominant and less abundant species. Integrating all these tools provided a thorough and reliable picture of the microbial community, ensuring accurate taxonomic assignments and precise abundance estimates from the shotgun metagenomic data.

### 2.7. Identification of ARGs and MGEs

Antimicrobial resistome analysis was performed by aligning the unigenes to the CARD database (v2.0.1) using blastp, with an e-value threshold of ≤1 × 10^−30^ to ensure high confidence and specificity in the annotation of ARGs from the metagenomic datasets. The abundance of antimicrobial resistance genes (ARGs) was calculated as fragments per kilobase per million fragments (FPKM) for contigs containing ARGs. The Resistance Gene Identifier (RGI) tool was used to analyze the alignment results, which were then used to assess the distribution of resistance genes across samples, the taxonomic sources of these genes, and the underlying resistance mechanisms.

### 2.8. Statistical Analysis

All statistical analyses were performed using Python (v3.11) with standard packages including pandas, NumPy, and SciPy. The data were first checked for normality. For normally distributed variables (e.g., body weight, biochemical parameters), one-way ANOVA followed by Dunnett’s post hoc test was used. For non-normal data (e.g., alpha diversity, ARG abundance), the Kruskal–Wallis test was applied, followed by Dunn’s multiple comparison test with Benjamini–Hochberg correction for multiple testing. Beta diversity was assessed using PERMANOVA based on Bray–Curtis dissimilarity. We reported the F-statistic, R^2^, and *p*-values for each comparison. LEfSe analysis was used to identify differentially abundant taxa, with a threshold of LDA > 2.0 and FDR-adjusted *p* < 0.05. Cohen’s d was calculated to estimate effect sizes for key features, such as alpha diversity, ARGs, and metabolic markers. The significance level of *p* < 0.05 was used throughout this study.

## 3. Results and Discussion

### 3.1. Effect of EF-2001 on Body Weight in HFD-Induced Obese Mice

**Figure 1:**
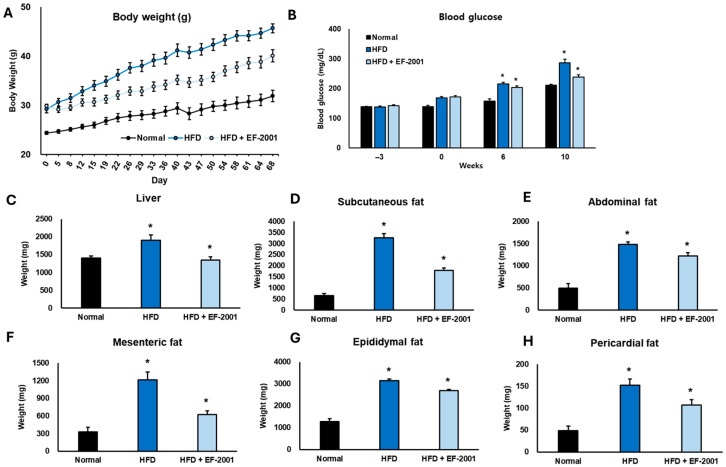
**Effects of EF-2001 on metabolic parameters in HFD-induced obese mice**. The animals had free access to food and water and were acclimated for three weeks before the experiment began (−3 weeks). (**A**) Body weight; (**B**) blood glucose levels; (**C**) organ weight and (**D**–**H**) fat depots were measured at corresponding weeks across three groups: ND diet, HFD, and HFD + EF-2001. EF-2001 supplementation significantly mitigated the rise in blood glucose and other adverse metabolic changes induced by HFD, particularly at week 10 (* *p* < 0.05 vs. the HFD group vs. the ND group). Data are presented as mean ± SEM.

At the start of this study, all the groups had similar body weights, but over time, significant differences were observed (Figure 1A). By Day 43 (6 weeks), the mice on an HFD had gained significantly more weight (~44 g), while those receiving EF-2001 had a noticeably lower weight (~38 g), showing a 13.64% reduction in weight gain (Figure S2). This pattern continued throughout this study, and by the final day (10 weeks), the HFD group weighed ~48 g, whereas the HFD + EF-2001 group weighed around 40 g, marking a 16.67% decrease compared to the HFD group. Interestingly, the HFD + EF-2001 group was much closer in weight to the ND group (~32 g) than to the HFD group, suggesting that EF-2001 helped counteract the effects of the HFD (Figure 1A). By the end of this study, the HFD group weighed 50% more than the ND group, while the HFD + EF-2001 group was only 25% heavier than the ND group, reinforcing the idea that EF-2001 plays a protective role in weight management (Figure S2). While it did not entirely prevent weight gain, it slowed it down, keeping body weight closer to normal. This is consistent with our previous research exploring the impact of EF-2001 administration on HFD-induced obese rats. EF-2001 significantly reduced body weight after six weeks in the obese rats [34]. Another study closely related to EF-2001 investigated the effects of *E. faecalis* EF-1, which mitigated HFD-induced body weight gain [14]. This finding further supports the potential of certain *E. faecalis* strains in managing body weight in obese mice. Similar findings indicated that supplementation with heat-killed *Lactobacillus brevis* KB290 significantly reduced body weight and fat accumulation in the HFD-induced mouse model [35]. These results suggest that EF-2001 could be a promising dietary supplement for managing obesity and maintaining a healthier body weight.

### 3.2. Effect of EF-2001 on Blood Glucose in HFD-Induced Obese Mice

We examined how EF-2001 affected blood glucose levels in the mice fed an HFD over 10 weeks (Figure 1B). Measurements were taken at the beginning (week 0), mid-point (week 6), and the end of this study (week 10). Before DIO, all three groups had similar blood glucose levels. However, the HFD-fed group already showed slightly higher levels (158.3 ± 5.6 mg/dL) compared to the ND group (135.2 ± 4.1 mg/dL), amounting to an increase of about 17%. The HFD mice administered EF-2001 had similar glucose levels (154.6 ± 5.2 mg/dL), suggesting no initial differences between the HFD diet groups (Figure 1B). At week 6, the impact of the HFD became more apparent. Blood glucose in the HFD group climbed to 206.4 ± 6.8 mg/dL, a 30% increase from 0 weeks. In contrast, the ND group saw only a slight rise (148.9 ± 4.8 mg/dL, about 10%). Interestingly, the HFD mice administered EF-2001 had glucose levels of 198.2 ± 6.1 mg/dL, about 4% lower than the untreated HFD group. Our previous findings observed a similar pattern: EF-2001 enhanced sensitivity to glucose, insulin, and leptin [34]. Although that study was conducted in rats, the reported improvement in glucose sensitivity provides a potential mechanism for the reduced blood glucose levels observed in our mouse study. Specifically, the probiotic *B. longum* APC1472 helped to normalize glucose levels, indicating increased tolerance in HFD mice [36]. This suggests that EF-2001 might slow the progression of glucose dysregulation. By week 10, the difference was even more striking. Blood glucose in the HFD group surged to 289.3 ± 8.4 mg/dL, an 83% jump from baseline, indicating severe glucose imbalance. However, EF-2001 supplementation significantly reduced this rise, with glucose levels reaching 230.1 ± 7.3 mg/dL, about 20% lower than the untreated HFD group (*p* < 0.05).

These results suggest that EF-2001 helps counteract the harmful effects of a high-fat diet on blood glucose levels. By slowing down glucose elevation, EF-2001 may play a protective role in preventing insulin resistance and metabolic disorders associated with obesity.

### 3.3. Effect of EF-2001 on Organ Weight in HFD-Induced Obese Mice

This study revealed significant organ and fat tissue weight changes among the experimental groups over the 10 weeks. The HFD led to substantial increases in fat accumulation compared to the ND, highlighting obesity-related effects. However, supplementation with EF-2001 in the HFD group effectively reduced organ and fat weights, particularly at the 6- and 10-week time points. Liver weight showed a noticeable reduction with EF-2001 treatment, decreasing by 20.0% at week 10 compared to the HFD group (Figure 1C). By week 10, the liver weight in the EF-2001-treated group was only 6.25% higher than in the ND group, suggesting a strong protective effect.

Subcutaneous fat also responded positively to EF-2001, showing a reduction by 28.57% at week 10 (Figure 1D). At the 10-week mark, subcutaneous fat in the HFD group was 85.71% higher than in the ND group, whereas EF-2001 treatment significantly reduced this excess. Abdominal fat followed a similar trend, decreasing by 20.0% at week 10 with EF-2001 (Figure 1E). At week 10, the HFD group had 60% more abdominal fat than the ND group, while the EF-2001 group showed an apparent reduction towards normal levels. Similarly, mesenteric fat weight decreased by 41.67% in week 10 with EF-2001 supplementation (Figure 1F). Compared to the ND group at week 10, mesenteric fat in the HFD group was 66.67% higher, while EF-2001 treatment significantly lowered this accumulation.

Epididymal fat, another major fat depot, was 16.67% lower at week 10 in the HFD + EF-2001 group compared to the HFD group (Figure 1G). By the 10th week, epididymal fat in the HFD group was 66.67% higher than in the ND group, while EF-2001 supplementation considerably reduced the excess fat accumulation. Pericardial fat also showed a moderate but meaningful reduction, decreasing by 26.67% in week 10 with EF-2001 (Figure 1H). At week 10, pericardial fat in the HFD group was 50.0% higher than in the ND group, whereas EF-2001 treatment brought this difference closer to normal levels (Figure 1H). These results are strongly supported by the previous literature, which indicates that EF-2001 treatment in DIO mice led to a decrease in fat mass and the liver index, as well as a significant reduction in epididymal adipose tissue weight [28]. Other findings reported that the administration of the probiotic *B. longum* subsp *infantis* FB3-14 significantly suppressed the gain in both body and fat weight [37].

Overall, the findings indicate that EF-2001 supplementation plays a crucial role in counteracting the effects of an HFD, with significant reductions in organ and fat weights over time. By the 10-week mark, the EF-2001 group showed values much closer to the ND diet group, reinforcing its potential in mitigating diet-induced obesity-related fat accumulation.

### 3.4. Effect of EF-2001 on Liver Function Markers in HFD-Induced Obese Mice

**Figure 2:**
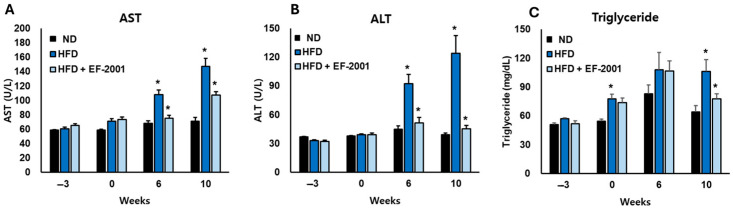
**Effects of EF-2001 on liver function markers**. The lipid profile (**A**) AST, (**B**) ALT and (**C**) Triglyceride were measured at weeks 0, 6, and 10 across the three groups: ND diet, HFD, and HFD + EF-2001. The animals had free access to food and water and were acclimated for three weeks before the experiment began (−3 weeks). (* *p* < 0.05 vs. the HFD group vs. the ND group). Data are presented as mean ± SEM.

The administration of EF-2001 demonstrated a strong protective effect against the metabolic disturbances induced by the HFD, particularly in liver function and lipid metabolism. The AST levels in the HFD group increased significantly by 50% in 6 weeks and 92% in 10 weeks compared to the ND group, indicating substantial liver stress (Figure 2A). However, EF-2001 supplementation effectively counteracted this increase, reducing AST levels by approximately 25% at 6 weeks and 30% at 10 weeks relative to the HFD group, bringing values closer to those observed in the ND group (Figure 2A).

A similar trend was observed in ALT levels, which spiked by 157% at 6 weeks and 266% at 10 weeks in the HFD group compared to the ND group, highlighting the extent of liver dysfunction. EF-2001 administration significantly lightened this elevation, leading to a 28% reduction at 6 weeks and a 40% reduction at 10 weeks compared to the HFD group. This suggests that EF-2001 plays a crucial role in preserving liver integrity and function despite prolonged exposure to an HFD (Figure 2B).

Triglyceride levels also showed a marked increase in the HFD group, with a 47% rise at 6 weeks and a 57% rise at 10 weeks compared to the ND group (Figure 2C). EF-2001 supplementation helped mitigate this effect, reducing triglyceride levels by 17% at 6 weeks and 23% at 10 weeks relative to the HFD group. Notably, the HFD + EF-2001 group consistently maintained values closer to the ND group across all measured parameters, reinforcing its potential to counteract HFD-induced metabolic disturbances. Several *Lactobacillus* strains, including *L. plantarum* [38], *L. paracasei* [39], *L. rhamnosus* [39], and *L. fermentum* [40], have demonstrated anti-obesity effects, including reductions in body weight, improvements in glucose tolerance and insulin sensitivity, and modulation of lipid metabolism and liver function. Some studies have also explored the effects of heat-killed *Lactobacillus* and *Bifidobacterium* strains, indicating that even non-viable bacteria can exert beneficial metabolic effects [41]. These findings suggest that heat-killed EF-2001 could serve as a promising intervention to mitigate diet-induced liver damage and lipid imbalances, offering potential therapeutic benefits for metabolic disorders associated with excessive fat consumption.

### 3.5. Metagenome Diversity Analysis

**Figure 3:**
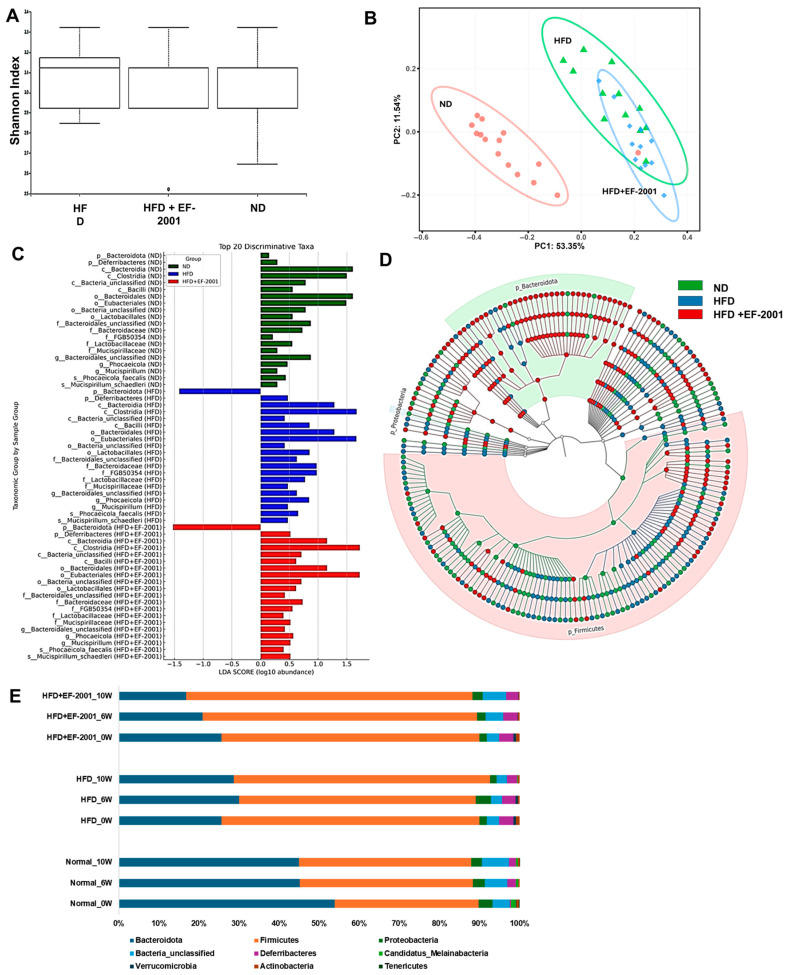
**Effect of EF-2001 on the gut microbiota in the HFD-induced obese model after 10 weeks of administration.** (**A**) Shannon alpha diversity. (**B**) Principal coordinate analysis (PCoA) based on Bray–Curtis dissimilarity. (**C**) LEFse analysis with a cutoff value of log10 (LDA score). (**D**) Taxonomic levels represented by rings, with phyla (Top 400) at the innermost ring and species at the outermost ring. Each circle is a member within that level. (**E**) Phylum level taxonomic distribution across all groups.

Alpha diversity analysis, measured by the Shannon index (Figure 3A), demonstrated that HFD feeding reduced gut microbial diversity compared to the ND group (Figure 3A). The HFD group’s reduced alpha diversity aligns with existing research indicating that a decrease in microbial richness is often associated with various health problems, including obesity [42]. Interestingly, EF-2001 supplementation in the HFD + EF-2001 group led to a higher alpha diversity than that in the HFD group alone, suggesting that the postbiotic had a restorative effect on microbial richness (Figure 3A). The boxplot further highlights that while the ND group had the highest microbial diversity, EF-2001 supplementation mitigated HFD-induced reductions in diversity (Figure 3A). However, an outlier in the HFD + EF-2001 group indicates some inter-individual variability in response to EF-2001 administration. Reduced microbial diversity is associated with gut dysbiosis, metabolic dysfunction, and inflammatory responses linked to obesity. The ability of EF-2001 to enhance alpha diversity suggests that this postbiotic might provide a broader range of substrates or create conditions that support the growth and survival of a more diverse microbial community, potentially counteracting the selective pressures imposed by the HFD [26,43].

Beta diversity, which assesses the differences in microbial community composition between samples, was analyzed using principal coordinate analysis (PCoA) based on Bray–Curtis dissimilarity (Figure 3B). The clear separation observed between the ND and HFD groups on the PCoA plot indicates that the high-fat diet substantially impacted the overall structure of the gut microbial community (Figure 3B). This finding is consistent with previous research, which showed that HFD significantly alters gut microbial composition in mice and pigs [44,45]. The distinct clustering of the HFD group away from the ND group visually confirms the profound effect of an HFD in driving the gut microbial community towards dysbiosis. The observation that the HFD + EF-2001 microbiome clustered between the ND and HFD groups suggests that EF-2001 induced a shift in the microbial community composition towards a healthier profile, partially reversing the changes caused by the HFD. This is supported by studies showing that postbiotics can modulate gut microbiota composition in the context of metabolic dysfunction. The intermediate clustering implies that EF-2001 dosing with 30 billion cells/day does not fully restore the microbiome to the healthy state observed in the ND group. Instead, it moves it in that direction, indicating a partial yet potentially beneficial modulation of the HFD-altered community [43].

### 3.6. Gut Microbiota Variability

The PERMANOVA analysis in this study revealed significant variability among the experimental groups, confirming that the HFD and EF-2001 supplementation interventions had a statistically significant impact on the overall microbial community structure (Figure S3) [45]. The HFD group displayed more significant variability in microbial composition, as evidenced by a wider interquartile range, while the HFD + EF-2001 group exhibited more consistent microbial profiles with reduced inter-individual variability (Figure S3). These results confirm that the microbiome composition significantly differed across the groups (e.g., HFD vs. HFD + EF-2001: F = 3.87, R^2^ = 0.31, *p* = 0.012). Notably, the ND group showed the most compact distribution, suggesting a stable microbial community structure [46]. The HFD + EF-2001 group, although sharing some similarity with the HFD microbiota due to the same HFD diet, demonstrated a divergent trend towards the ND group. This indicates that EF-2001 supplementation modulates the gut microbiome, nudging it away from the dysbiotic state induced by the HFD and towards a composition more closely resembling that of the healthy control group. This trend, coupled with the reduced variability within the HFD + EF-2001 group, suggests that the postbiotic not only shifted the average microbial composition but also promoted a more uniform and potentially beneficial response at the individual level.

### 3.7. Gut Microbiota at the Phylum Level

The gut microbiota comprises eight to nine major phyla, with *Firmicutes* and *Bacteroidota* being the most abundant [47]. High-fat diet (HFD) feeding is known to decrease the relative abundance of Bacteroidota while increasing the abundance of Firmicutes and Proteobacteria [48,49]. In the present study, both the HFD and HFD + EF-2001 groups exhibited reduced Bacteroidota and increased Firmicutes compared to the ND group, confirming the typical impact of a high-fat diet on these dominant phyla (Figure S4). The Lefse analysis revealed the differences in classification characteristics at different levels (Figure 3C,D).

Interestingly, EF-2001 administration induced a transient increase in *Bacteroidota* and a slight decrease in *Firmicutes* during the first six weeks (Figure 3E). This transient effect is reminiscent of the effects often observed with probiotic administration on the gut microbiota [50,51]. Previous studies also highlight the potential of postbiotics to influence microbial communities in the gut [52,53]. This initial increase in *Bacteroidota* might suggest that EF-2001 initially provides a more favorable environment or specific nutrients that promote the growth of this phylum. However, by week 10, *Bacteroidota* levels declined again along with increasing *Firmicutes* levels, as shown in Figure 3E. This suggests a long-term adaptation of gut microbial composition due to the HFD diet and insufficient EF-2001 supplementation to modulate it toward a healthy gut microbiome. EF-2001 can exert an initial influence on the dominant phyla; the sustained presence of the HFD might eventually lead to a partial return to the phylum balance typically associated with a high-fat diet. Additionally, *Proteobacteria* remained consistently lower in the HFD + EF-2001 group compared to both the HFD and ND groups, further indicating a stabilizing effect of EF-2001 on gut microbial balance (Figure 3E). This reduction in Proteobacteria by EF-2001 could be a significant benefit, as an increase in this phylum is often associated with gut dysbiosis and inflammation [54]. Many opportunistic pathogens belong to the Proteobacteria phylum, and their overgrowth is frequently linked to various gut-related disorders and diseases [55]. Therefore, the ability of EF-2001 to maintain lower levels of Proteobacteria suggests a potential stabilizing effect on the gut microbial balance and a possible mechanism for mitigating the inflammation associated with an HFD.

Overall, at the phylum level, the findings align with prior research demonstrating that postbiotics, like EF-2001, can reshape gut microbial communities, enhance microbial diversity, and restore microbial equilibrium in obesity-associated dysbiosis, ultimately contributing to improved host metabolic health [56,57].

### 3.8. Genus Level Analysis

**Figure 4:**
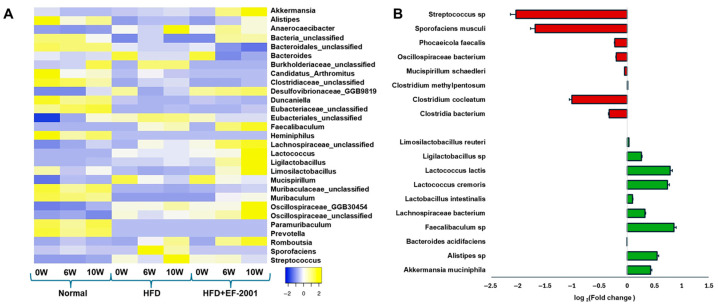
**Gut microbial distribution among different fecal samples**. (**A**) Heat map showing the microbial distribution among three samples at different timepoints at the genus level (top 30 taxa). (**B**) Fold change after EF-2001 administration after 10 weeks (HFD vs. HFD + EF-2001).

Genus-level analysis revealed a significant increase in several beneficial bacterial genera in the HFD + EF-2001 group compared to the HFD group, including *Akkermansia*, *Alistipes*, *Muribaculum*, *Faecalibaculum*, *Lactococcus*, *Ligilactobacillus*, *Limosilactobacillus*, and *Lachnospiraceae* (Figure 4A). Notably, *Akkermansia*, *Alistipes*, *Ligilactobacillus*, and *Lachnospiraceae* were more abundant in the HFD + EF-2001 group than in the ND group, suggesting a potential role in gut barrier function and mucosal immunity enhancement.

### 3.9. Species-Level Analysis: HFD vs. HFD + EF-2001

The species-level analysis revealed significant microbial shifts between the HFD and HFD + EF-2001 groups at 10 weeks (Figure 4B). The changes observed in bacterial populations highlight the impact of EF-2001 supplementation on gut microbiota composition, particularly in terms of beneficial and pathogenic bacteria. Postbiotics like EF-2001 can contribute to a balanced gut environment by inhibiting pathogen growth and promoting beneficial bacterial growth [52]. Another study reported that *Bacillus velezensis* cell-free supernatant in HFD-induced obese mice resulted in significant changes in the gut microbiota, notably an enrichment in SCFA-producing bacteria, such as Roseburia and Eubacterium [24]. This can occur through various mechanisms, including the production of SCFAs and bacteriocins, which can directly inhibit pathogen growth [23,58]. Beneficial bacteria that contribute to gut health by producing SCFAs, such as butyrate, propionate, and acetate, were significantly increased in the HFD + EF-2001 group compared to the HFD group alone. These SCFAs are crucial in maintaining gut integrity, modulating inflammation, and regulating metabolism [59]. *A. muciniphila*, a well-established beneficial bacterium, improves gut barrier integrity, reduces inflammation, and enhances SCFA production [60]. Its 0.44-fold change after 10 weeks of EF-2001 administration suggests a protective role against HFD-induced dysbiosis (Figure 4B). The rise of 54% (0.26-fold) in Ligilactobacillus was observed in the HFD + EF-2001 group compared to the HFD group at 10 weeks (Figure 4B and Figure S5A). This genus contributes to SCFA production, which is particularly beneficial in counteracting the inflammatory effects of an HFD [23]. Ligilactobacillus species are also known for their roles in maintaining intestinal barrier integrity, modulating immune responses, and producing antimicrobial compounds that inhibit pathogen colonization. An increase in Ligilactobacillus reinforces the trend of EF-2001 promoting SCFA-producing bacteria, which is crucial for mitigating the adverse effects of an HFD. This functional shift suggests that EF-2001 may help re-establish microbial balance and support gut metabolic health under dietary stress. Other probiotics and postbiotics have been shown to increase the abundance of beneficial bacteria, like *A. muciniphila* [60] and Lactobacillus [61], and decrease potentially harmful bacteria [26]. Previous research indicates that EF-2001 supplementation can lead to specific changes in the abundance of certain bacterial taxa. For instance, in a study on HFD-induced obese mice, heat-killed *E. faecalis* EF-2001 attenuated lipid accumulation [28]. While the study focused on metabolic outcomes, changes in gut microbiota composition likely contributed to these effects. These findings suggest that EF-2001, as a postbiotic, may exert similar selective pressures on the gut microbiota, favoring the growth of beneficial species and suppressing detrimental ones.

*Lactococcus lactis* and *Lactococcus cremoris*, commonly used in dairy fermentation, may influence gut microbiota composition and metabolite profiles, potentially contributing to improved gut barrier function [62] and reducing gut permeability issues caused by an HFD. The increase in Lactococcus by ~16% (Figure S5A), a genus often used in probiotics, suggests that EF-2001 might promote bacterial growth with direct benefits for gut barrier integrity and metabolic function [63]. Recent studies suggest that *Lactococcus* may benefit obesity management by reducing fat accumulation in mice [64,65]. A similar trend was observed with the administration of *L. lactis* subsp. *cremoris*, which effectively mitigated metabolic changes induced by a Western-style diet in a mouse model [66]. This is consistent with our in vivo studies showing that body weight and blood glucose levels were significantly reduced after 10 weeks of EF-2001 administration (Figure 1). Few lactic acid postbiotics have been reported to enhance SCFA production by modulating gut microbiota. Additionally, they have shown potential in improving NAFLD, regulating glucose metabolism, and enhancing insulin sensitivity [67,68]. For instance, *Lactiplantibacillus plantarum* LRCC5314 has been shown to modulate gut microbiota composition, contributing to a significant reduction in insulin resistance and fat accumulation [69]. Other beneficial bacteria, including *Alistipes* sp., *Bacteroides acidifaciens*, *Faecalibaculum* sp., *Lactobacillus intestinalis*, and *Lachnospiraceae* bacterium, exhibited a fold change following 10 weeks of EF-2001 administration. Notably, *Faecalibaculum* sp. showed a pronounced increase (0.86-fold or 86%) in the HFD + EF-2001 group compared to the HFD group (Figure 4B and Figure S5A). Its known association with SCFA production, particularly butyrate, suggests a potential role in mitigating inflammation and promoting gut microbiota homeostasis [70]. Similarly, *Alistipes* sp. has been implicated in SCFA production and has demonstrated potential benefits in metabolic disorders, including obesity, further supporting the beneficial modulatory effects of EF-2001 on gut microbial composition [71]

Conversely, several opportunistic pathogenic bacteria were significantly reduced in the HFD + EF-2001 group, suggesting that EF-2001 supplementation may help mitigate the harmful effects of HFD-induced microbiome disturbances. The most notable reductions include Streptococcus sp., which is associated with pro-inflammatory responses and metabolic disorders, indicating a potential shift towards a healthier gut environment [72]. *Sporofaciens musculi*, which does not have any protective characteristics in certain conditions [73], showed a marked 1.69-fold decrease (99%) at week 10 (Figure 4B), which may contribute to improved metabolic function and a healthy gut. While certain members of the *Oscillospiraceae* family exhibit beneficial properties, specific species have been implicated in the potential pathogenesis of obesity [74]. Conversely, unclassified *Oscillospiraceae* was reduced 43% (0.2-fold) in individuals subjected to HFD + EF-2001 (Figure S5B), demonstrating a positive correlation with serum cholesterol levels [75]. *Phocaeicola faecalis* and *Mucispirillum schaedleri*, known for their ability to thrive in an inflamed gut, showed a reduction over 10 weeks (0.2 fold), which suggests a potential anti-inflammatory effect of EF-2001 (Figure 4B). While SCFAs generally have beneficial roles in gut health, some studies suggest that specific Clostridium species can be elevated in the context of metabolic disorders. For instance, in a mouse model of NAFLD, particular Clostridium species were found to be increased [76]. *Clostridium cocleatum* has been implicated in gut dysbiosis and inflammatory processes [77]. Some studies have shown increased *C. cocleatum* abundance in animal models of metabolic disorders induced by an HFD. Notably, one study found that *C. cocleatum* abundance increased significantly in high-fat diet-fed mice treated with metformin, which improved metabolic disorder markers [78]. However, other studies linked elevated levels of certain Clostridium species with pro-inflammatory conditions and liver diseases, including NAFLD [76]. The observed reduction in Clostridium species by a 1-fold change (Figure 4B) in the HFD + EF-2001 group might suggest a beneficial effect of EF-2001 in mitigating potential pro-inflammatory aspects associated with this species in the context of an HFD.

### 3.10. ARG Abundance

**Figure 5:**
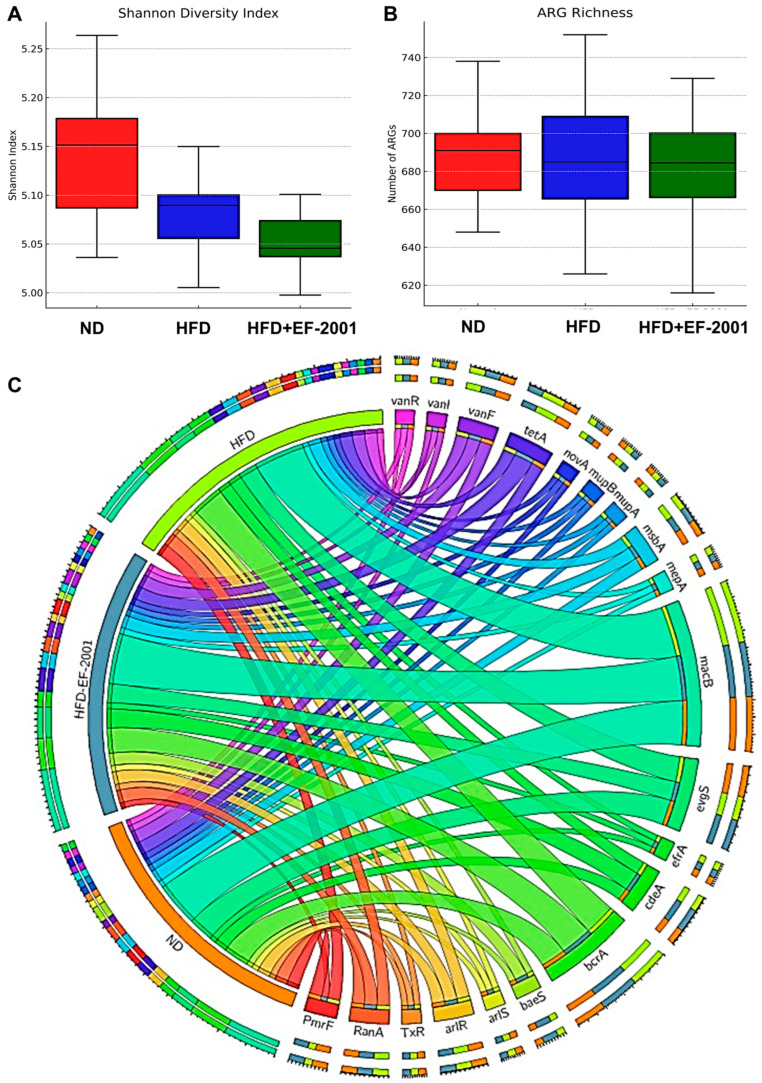
**Abundance and distribution of ARGs among the different treatment groups.** (**A**) Shannon and (**B**) richness indices of the ARGs among the different groups. (**C**) CARD analysis for ARG distribution (Top 20) in the microbial phyla.

The Shannon index and ARG richness indicated a clear distinction between the HFD and ND groups, highlighting the impact of the HFD in this study (Figure 4B and Figure 5A). The analysis of the top 20 ARGs revealed distinct variations in their relative abundances across different conditions: ND, HFD, and HFD + EF-2001 (Figure 5C). The most abundant ARG subtype observed was *mac*B, which exhibited a minor increase in the HFD group (7.55%) compared to the ND group (7.38%) and remained stable in the HFD + EF-2001 group (7.53%) (Table S1). The prevalence of *mac*B is further highlighted by its ranking among the top five most abundant ARGs in the gut resistome of the plateau pika [79]. Multidrug resistance-associated ARGs showed variable trends, including *bcr*A, *evg*S, and *cde*A. Efflux pumps, like BcrA, play a crucial role in bacterial survival by lowering the intracellular concentration of antibiotics and other toxic compounds [80]. In *Lachnoclostridium*, the abundance of *bcr*A is influenced by diet, with higher levels observed in control and phytogenic feed additive-treated groups compared to antibiotic-treated groups [32]. In the current study, *bcr*A exhibited a modest increase from 4.32% in the ND group to 4.96% in the HFD group and decreased to 4.88% in the HFD + EF-2001 group (Table S1). This trend suggests that both the HFD and the EF-2001 supplementation may contribute to the enrichment in bacteria utilizing the BcrA efflux pump. Conversely, *evg*S remained unchanged across all conditions, suggesting its abundance is less influenced by dietary variations. The increased abundance of the efflux pump gene *cdeA* in response to the HFD may reflect a bacterial adaptation to stress-associated metabolites elevated by the diet [81]. This adaptation could be linked to the need to expel compounds such as bile acids and fatty acid metabolites, which are known to exert antimicrobial effects and influence microbial survival in the gut environment. The variable trends observed across all conditions suggest that the regulation of *cdeA* might be sensitive to various environmental factors, including dietary composition.

Several ARGs associated with antibiotic resistance mechanisms (e.g., *msbA*, *RanA*, *arlR*, the *vanR* gene in the *vanF* cluster) demonstrated moderate increases in HFD conditions, with some further slight decreases in the HFD + EF-2001 group (Figure 5C). However, a few ARGs, such as *TxR* and *mupB*, exhibited a slight decreasing trend in response to EF-2001 supplementation (Table S1), which may indicate a selective effect on certain resistance determinants. The moderate increase in *msbA* with the HFD could be related to changes in the Gram-negative bacterial population within the gut or an upregulation of this efflux pump in response to the metabolic stress or altered membrane composition associated with an HFD [82]. The increase in *arlR* abundance in response to the HFD might suggest a subtle increase in the population of Staphylococcus or other bacteria that utilize this regulatory system [83]. The subsequent slight decrease with EF-2001 supplementation indicates a potential influence of the postbiotic on these bacterial populations or the regulation of the *arlR* gene. It was noted that all top 20 ARGs belong to either Firmicutes or Proteobacteria, except for the macrolide subgroup (Table S1). EF-2001 may shift microbial composition away from ARG-harboring taxa (e.g., Proteobacteria) and potentially suppress ARG expression through immune modulation or bacteriocin-like activity. There were no noticeable changes in the EF-2001-origin ARGs after the administration of EF-2001, indicating no significant horizontal gene transfer from EF-2001 to the gut microbiome (Table S2).

The effect of EF-2001 supplementation on ARG abundance appears to be gene-specific. While most ARGs remained relatively stable or increased slightly, genes such as *TxR* and *mupB*, which are associated with tetracycline resistance mechanisms and mupirocin resistance, respectively, showed a downward trend in the HFD + EF-2001 group [84,85]. This finding suggests that specific probiotic strains may modulate the prevalence of certain resistance genes, potentially through competitive exclusion or changes in microbial community dynamics [86].

EF-2001 is a commercially available postbiotic powder from bereum Co., Ltd., that contains heat-killed *E. faecalis* [87,88]. The existing research on heat-killed lactic acid bacteria, including *E. faecalis*, demonstrates their immunomodulatory effects and potential to protect against inflammatory bowel disease in animal models [89]. Furthermore, studies have shown that heat-killed *E. faecalis* EF-2001 can attenuate lipid accumulation in diet-induced obese mice [28]. The broader literature on various postbiotics, as highlighted in [26], generally supports their positive effects on gut microbial diversity, composition, and metabolic health in animal models. Additionally, analysis of antibiotic resistance genes (ARGs) revealed notable shifts in abundance across different conditions. Notably, HFD consumption led to an overall increase in several ARGs associated with multidrug resistance (e.g., *bcrA*, *msbA*, *baeS*, *efrA*), antibiotic efflux pumps (*macB*, *cdeA*, *tetA* (58), *vanR*), and specific antibiotic classes, such as fluoroquinolones (*arlR*, *arlS*), tetracyclines (*TxR*), and vancomycin resistance (*vanR* gene in *vanF* and *vanI* clusters) (Figure 5C and Figure S6). Notably, msbA and TxR exhibited a large effect size (Cohen’s d = 1.27) and statistically significant reductions when comparing the HFD and HFD + EF-2001 groups, suggesting a potential modulatory effect of EF-2001 on ARG prevalence. However, EF-2001 administration appeared to mitigate some of these increases, as observed in the reduced abundance of key resistance genes, such as *PmrF*, *mupA*, *novA, vanR*, and *TxR*, in the HFD + EF-2001 group compared to the HFD group (Figure 5C and Figure S6). *E. faecalis* EF-2001, likely the source of the postbiotic used in this study, has been characterized as lacking specific genes related to drug resistance and pathogenesis [90]. Furthermore, *E. faecalis* EF-2001 has shown susceptibility to a broad range of antibiotics, except for some aminoglycosides, suggesting that it is not inherently a reservoir of diverse ARGs [88]. Additionally, some postbiotics contain or stimulate the production of antimicrobial compounds, such as organic acids and bacteriocins, which can directly inhibit the growth of a broad range of bacteria, including those that have acquired antibiotic resistance [91]. For example, heat-killed *E. faecalis* has been shown to prevent the intestinal colonization of vancomycin-resistant enterococci in chicks [92]. Furthermore, postbiotics can modulate gene expression within the gut microbiota [93]. This includes the potential to downregulate the expression of antibiotic resistance genes [94]. These findings collectively suggest that the beneficial effects of EF-2001 observed in this study are consistent with the broader understanding of how postbiotics impact gut health and metabolic parameters.

This study provides important insights into the potential of heat-killed EF-2001 as a promising strategy to restore gut balance and mitigate diet-related metabolic disturbances. However, we recognize several limitations that also present valuable directions for future research. The sample size was determined based on the previous literature and practical constraints; nevertheless, future studies involving larger cohorts will help to enhance statistical power and improve the generalizability of our findings. The duration of this study was sufficient to observe notable microbial and physiological changes, laying a strong foundation for understanding the underlying mechanisms. Future research with extended follow-up periods and the integration of broader multi-omics approaches could further elucidate the long-term and systemic effects of EF-2001. Importantly, while our metagenomic analysis revealed significant modulation of antibiotic resistance genes (ARGs), we acknowledge that further meta-transcriptomic or functional validation assays (e.g., qPCR, phenotypic resistance profiling) are necessary to confirm the biological significance and activity of these changes. Overall, despite these limitations, our findings highlight the potential of heat-killed EF-2001 in improving gut health and metabolic outcomes in high-fat diet-fed mice, contributing valuable knowledge to the field and informing future mechanistic and translational studies.

## 4. Conclusions and Future Perspectives

This study demonstrates that an HFD disrupts gut microbial balance, increases antibiotic resistance genes ARGs, and contributes to metabolic disorders. EF-2001 supplementation restored gut homeostasis by enhancing microbial diversity, strengthening the gut barrier, and reducing inflammation. It also improved metabolic markers, including lower body fat, triglyceride levels, and liver enzyme activity. Notably, EF-2001 mitigated the HFD-induced increase in ARGs, particularly those linked to antibiotic efflux, suggesting its role in limiting antibiotic resistance. These findings highlight EF-2001 as a promising microbiome-targeted intervention for improving metabolic health and reducing antibiotic resistance risks. Future research should focus on the functional implications of these genes and their interactions with dietary components and postbiotics.

## Data Availability

The original contributions presented in this study are included in this article/Supplementary Material. Further inquiries can be directed to the corresponding author.

## Supplementary Materials

The following supporting information can be downloaded at https://www.mdpi.com/article/10.3390/bioengineering12070741/s1: Table S1. Representative ARGs subtypes and their abundance in each group after 10 weeks of EF-2001 administration. Table S2. *E. faecalis* strain specific ARGs subtypes and their abundance in each group after 10 weeks of EF-2001 administration. Figure S1. Study flow for three different diet groups. Figure S2. Effects of EF-2001 on body weight in HFD-induced obese mice. Figure S3. Box plots showing the significant difference concerning fold differential abundance of taxa identified in the gut microbiomes of Normal (ND), HFD and HFD+EF-2001 fed mice. Figure S4. Firmicutes/Bacteroidetes (F/B) ratio in three different groups at different time points. Figure S5. Comparison of relative taxa abundance distribution (species level) between HFD and HFD+EF-2001 groups after 10 weeks of EF-2001 administration. Bar graph shows significant changes in beneficial (A) and pathogenic (B) taxa. Figure S6. Heatmap shows ARGs distribution across various time points in different diet groups.
