# Supplementary material for: Modulation of Gut Microbiota and Antibiotic Resistance Genes by Heat-Killed Enterococcus faecalis EF-2001 in High-Fat Diet-Induced Obesity Mice: A Shotgun Metagenomics Study
Source: Bioengineering (Basel). 2025 Jul 7;12(7):741. doi: 10.3390/bioengineering12070741 (PMC12292747; doi:10.3390/bioengineering12070741)
Supplement: Supplementary file 1 [file bioengineering-12-00741-s001.zip › bioengineering-3679741-supplementary.pdf]

Modulation of Gut Microbiota and Antibiotic Resistance Genes by Heat-Killed *Enterococcus faecalis* EF-2001 in High-Fat Diet-Induced Obesity Mice: A Shotgun Metagenomics Study

Ranjith Kumar Manoharan[a]†, Kwon-Il Han[a], Hyun-Dong Shin[a], Yura Lee[a], Sunhwa Baek[a], Eunjung Moon[a], Youn Bum Park[a], Junhui Cho[a], Sathiyaraj Srinivasan [b†]

[a]Research & Development Center, Bereum Co., Ltd., Wonju 26361, Republic of Korea

[b]Department of Bio & Environmental Technology, College of Natural Science, Seoul Women's University, 623 Hwarangno, Nowon-gu, Seoul 01797, Korea

†Corresponding author: E-mail:mrkumarbiotech@gmail.com; drsrini@swu.ac.kr

Table S1. Representative ARGs subtypes and their abundance in each group after 10 weeks of EF-2001 administration

Sample No.	ARG Subtype	ARG Type	Resistance Mechanism	Abundance (percentage)			Taxa (Phylum)
				ND	HFD	HFD+EF-2001	
1	*macB*	Macrolide	Antibiotic efflux	7.38	7.55	7.53	Bacteroidetes
2	*bcrA*	Multidrug	Antibiotic efflux	4.32	4.96	4.88	Firmicutes
3	*evgS*	Multidrug	Regulatory system	3.53	3.51	3.47	Proteobacteria
4	*cdeA*	Multidrug	Antibiotic efflux	2.12	2.49	2.43	Proteobacteria
5	*tetA(58)*	Tetracycline	Antibiotic efflux	1.94	2.30	2.31	Proteobacteria
6	*msbA*	Multidrug	ABC transporter protein	1.84	2.25	2.15	Proteobacteria
7	*RanA*	Aminoglycoside	ABC-type efflux	1.72	2.16	2.04	Proteobacteria
8	*arlR*	Fluoroquinolone	Virulence and biofilm formation	1.95	1.86	1.84	Firmicutes
9	*vanR (vanF)*	Vancomycin	Antibiotic efflux	1.71	1.98	1.85	Firmicutes
10	*PmrF*	Polymyxin	Antibiotic resistance	1.87	1.57	1.44	Proteobacteria
11	*baeS*	Multidrug	Antibiotic efflux	1.15	1.44	1.37	Proteobacteria
12	*mepA*	Glycylcycline	Antibiotic efflux	1.09	1.22	1.18	Firmicutes
13	*vanR (vanI)*	Vancomycin	Antibiotic efflux	1.03	1.20	1.11	Firmicutes
14	*arlS*	Fluoroquinolone	Antibiotic efflux	1.14	1.11	1.07	Firmicutes
15	*mupA*	Mupirocin	Antibiotic resistance	1.10	1.07	1.03	Firmicutes
16	*novA*	Aminocoumarin	Antibiotic resistance	0.98	1.12	1.05	Firmicutes
17	*vanR*	Vancomycin	Antibiotic resistance	0.95	1.07	1.01	Firmicutes
18	*efrA*	Multidrug	Antibiotic efflux	0.91	1.03	0.97	Firmicutes
19	*TxR*	Tetracycline	Regulatory system	1.12	0.91	0.84	Firmicutes
20	*mupB*	Mupirocin	Antibiotic resistance	1.11	0.91	0.84	Firmicutes

*No significant changes were seen between HFD and HFD+EF-2001 groups after 10 weeks

Table S2. *E. faecalis* strain specific ARGs subtypes and their abundance in each group after 10 weeks of EF-2001 administration

Sample No.	ARG Subtype	ARG Type	Resistance Mechanism	Abundance (percentage)		
				ND	HFD	HFD+EF-2001
1	*dfrA*	Multidrug	Antibiotic efflux	0	0	0
2	*efrA*	Multidrug	Antibiotic efflux	0.91	1.03	0.97
3	*efrB*	Multidrug	Antibiotic efflux	0.43	0.54	0.51
4	*emeA*	Multidrug	Antibiotic efflux	0.02	0.03	0.02
5	*lsaA*	Macrolide-lincosamide-streptogramin	Antibiotic efflux	0.02	0.04	0.02

*These ARGs were identified in EF-2001 strains [1].

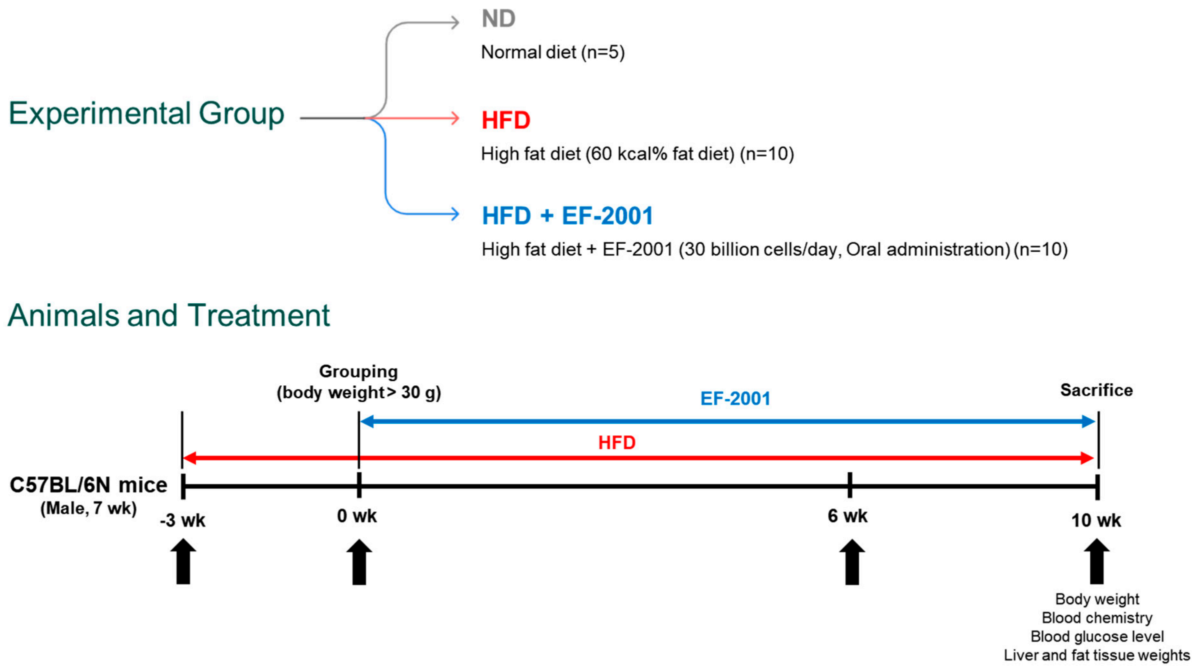

Figure S1. Study flow for three different diet groups

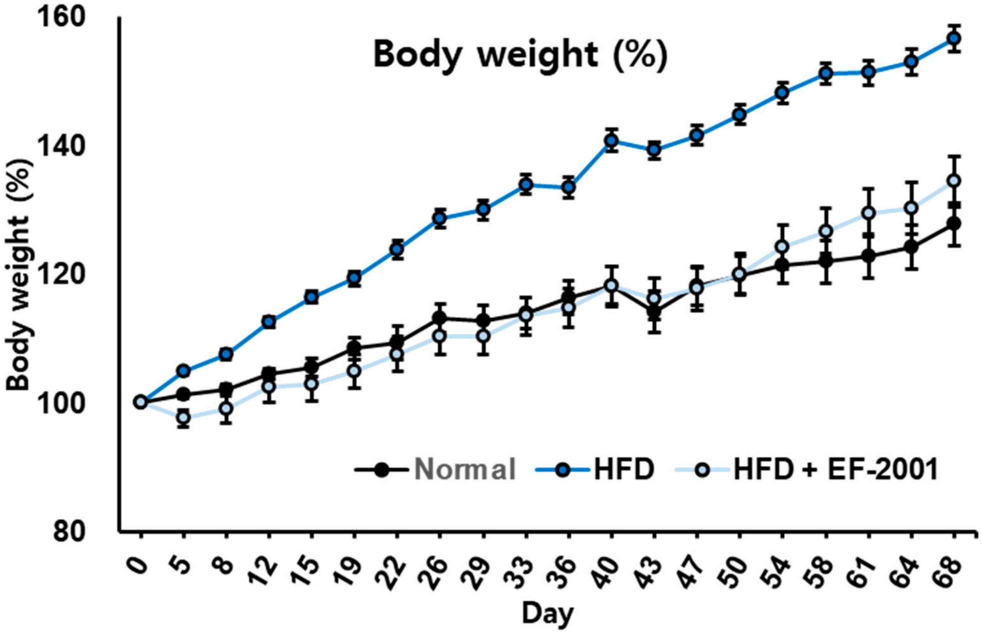

Figure S2. Effects of EF-2001 on body weight in HFD-induced obese mice

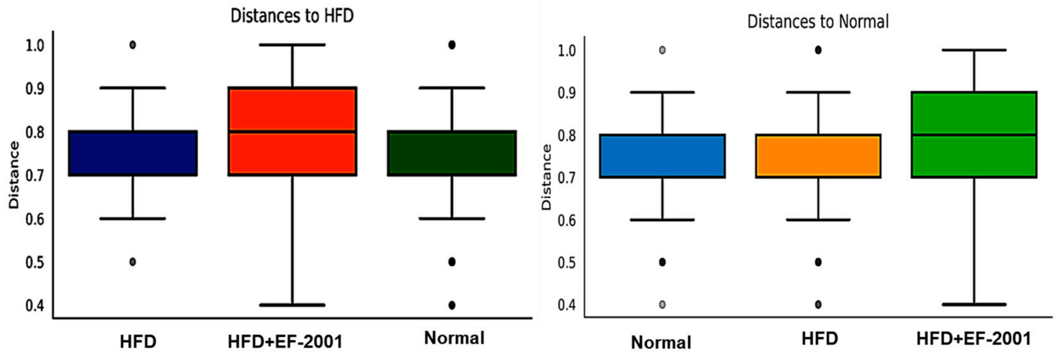

Figure S3. Box plots showing the significant difference concerning fold differential abundance of taxa identified in the gut microbiomes of Normal (ND), HFD and HFD+EF-2001 fed mice.

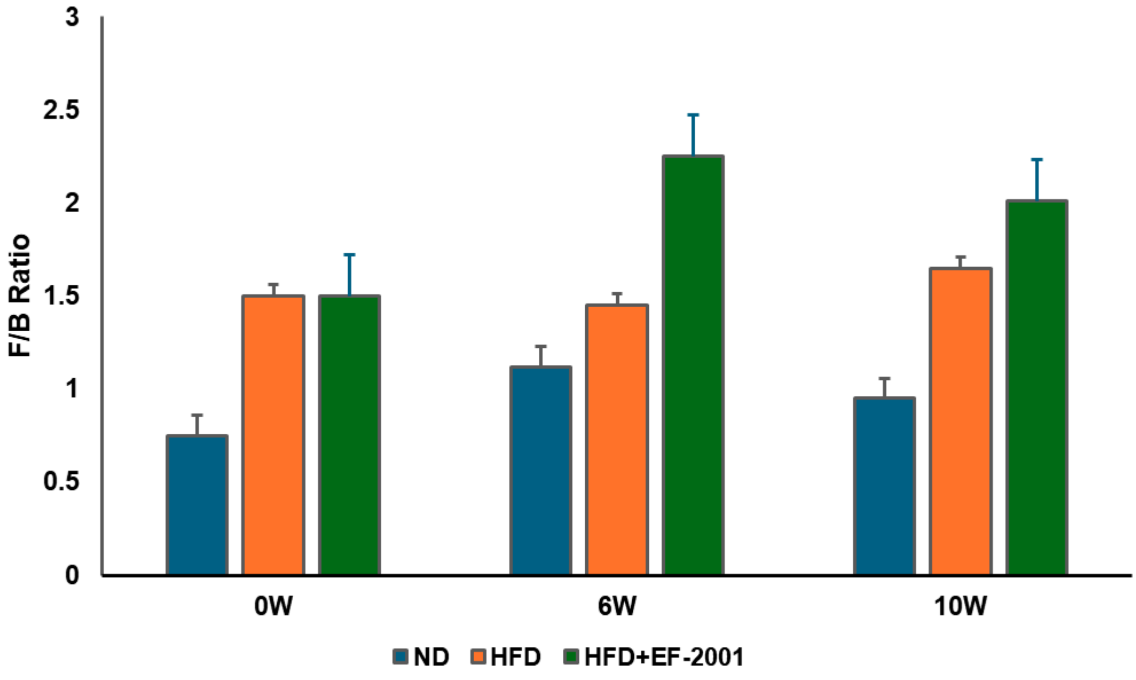

Figure S4. Firmicutes/Bacteroidetes (F/B) ratio in three different groups at different time points

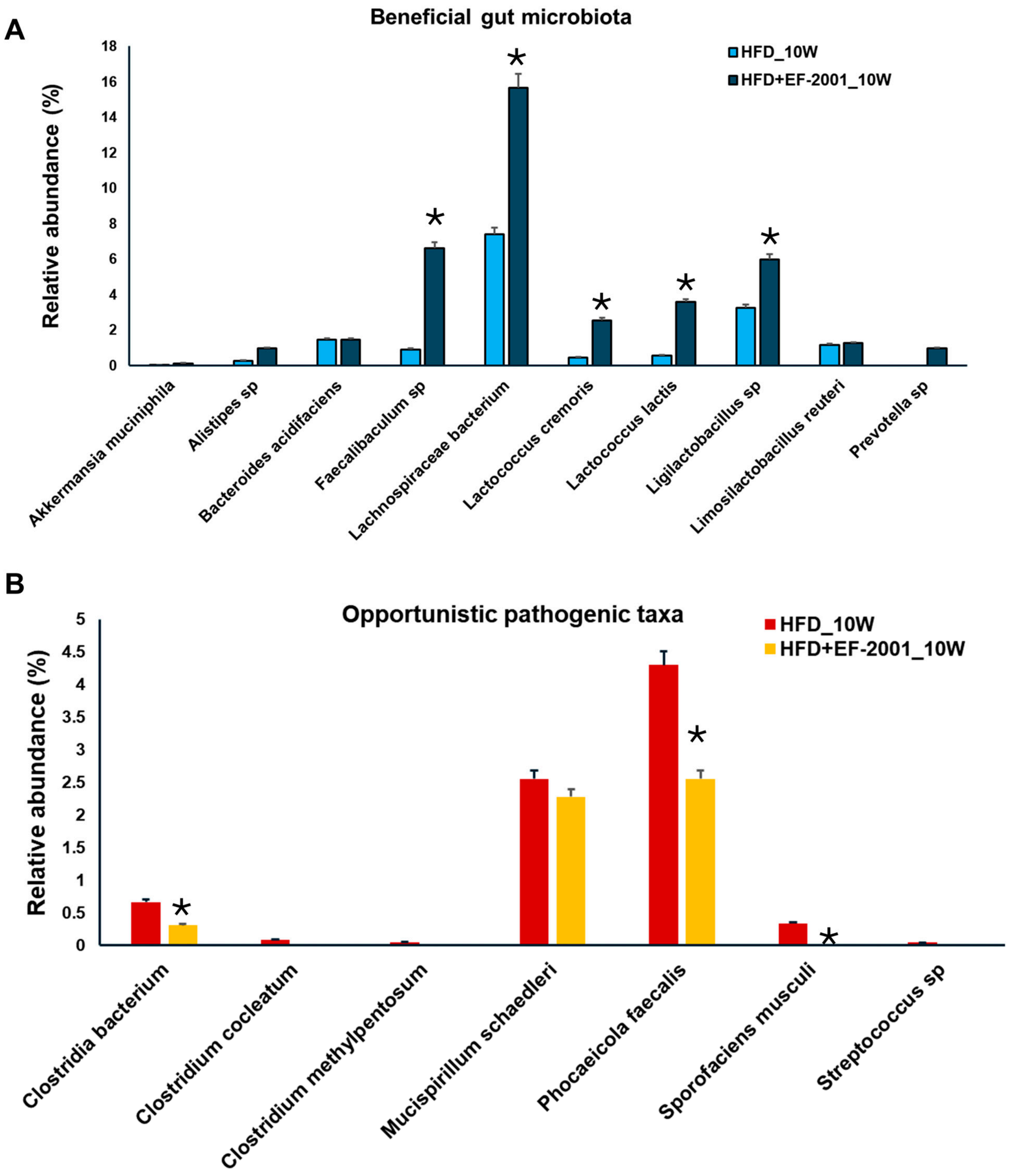

Figure S5. Comparison of relative taxa abundance distribution (species level) between HFD and HFD+EF-2001 groups after 10 weeks of EF-2001 administration. Bar graph shows significant changes in beneficial (A) and pathogenic (B) taxa.

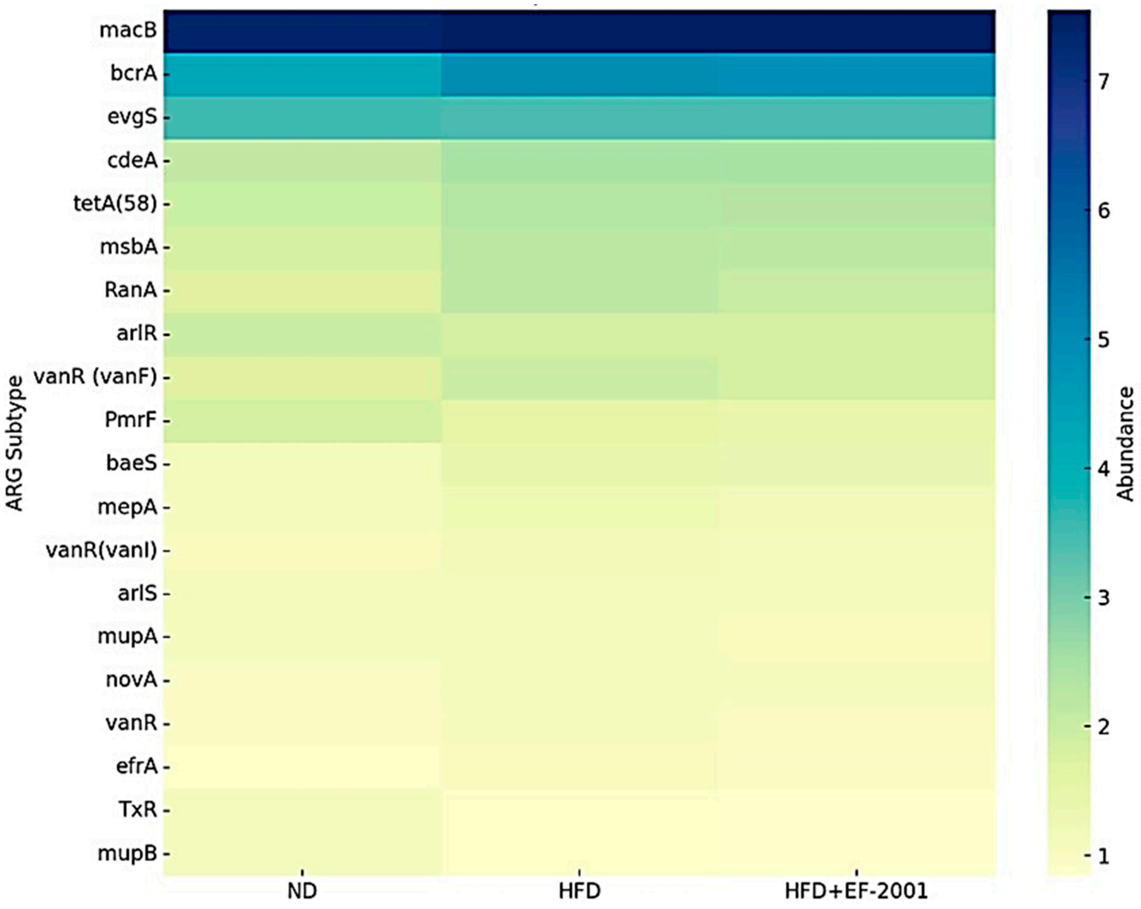

Figure S6. Heatmap shows ARGs distribution across various time points in different diet groups

References

1. Panthee, S.; Paudel, A.; Hamamoto, H.; Ogasawara, A. A.; Iwasa, T.; Blom, J.; Sekimizu, K., Complete genome sequence and comparative genomic analysis of *Enterococcus faecalis* EF-2001, a probiotic bacterium. *Genomics* **2021,** 113, (3), 1534-1542.
